# 

**Published:** 1904-01-23

**Authors:** 


					j.nA xiuariTAL. "The standard of highec. 4 ?.
CADBURY'S %X te* CADBURY'S
COCOA
NO DRUGS OR ALKALIES USED.
No. 904. Yol. XXXV. Saturday, Jan. 23rd, 1904. [Su6,cSyg""'] PRICE THREEPENCE.

				

